# Successful rehabilitation after multiple severe complications following orthognathic surgery: a case report

**DOI:** 10.1186/s12903-023-03644-1

**Published:** 2023-11-22

**Authors:** Cecilia Koskinen Holm, Lena C. Johansson, Malin Brundin, Mats Sjöström

**Affiliations:** 1 Division of Oral and Maxillofacial Surgery, Department of Odontology, Umeå University, Umeå, Sweden; 2 Wallenberg Centre for Molecular Medicine, Umeå University, Umeå, Sweden; 3 Prosthodontic Specialist Clinic, Region of Västerbotten, Umeå, Sweden; 4 Division of Endodontics, Department of Odontology, Umeå University, Umeå, Sweden

**Keywords:** Orthognathic surgery, Complications, Soft tissue, Vascularization, Necrosis

## Abstract

**Background:**

Complications of orthognathic surgery are quite rare, but they cause suffering in affected individuals. The range of complications is broad and includes both hard and soft tissue.

**Case presentation:**

We here present a case of a fully healthy woman without signs of impaired healing capacity. The patient underwent bimaxillary orthognathic surgery and experienced multiple complications both peri- and post-operatively. During the post operative period, the patient also suffered from soft tissue complications after an orthopaedic injury. Therefore, we referred the patient to her general practitioner for further medical investigation. We also present the result after restorative surgery and endodontic and prosthodontic treatment resulting in a successful rehabilitation.

**Conclusion:**

This case report clearly shows the need for a good collaboration between different odontological and medical fields to achieve a good and predictable result. In situations where normal healing processes do not occur, in-depth analysis must be carried out.

**Highlights:**

Orthognathic surgery affects soft and hard tissue which can result in adverse healing and complications. It is of great importance to follow up performed surgery to see late complications. Be restrictive with early re-operations when there are signs of necrosis. Always use a multidisciplinary approach when handling complications after surgery.

## Introduction

Orthognathic surgery is performed to correct primarily functional but also aesthetic alterations in the maxillofacial area. In Sweden, approximately 1 person in 10,000 per year undergoes orthognathic surgery [1], while in the US about 1 in 30,000 per year undergoes such surgery [2]. Depending on the specific indications for surgery, the surgical approach involves either or both the maxilla and mandible [3]. The most common mandibular approach is a bilateral sagittal split-osteotomy (BSSO) [3, 4] and Le Fort I-osteotomy (LF I) with or without segments in the maxilla [3, 4].

Although orthognathic surgery is a safe procedure, complications do occur [3–5]. The reported negative outcomes can be categorized as common or rare and subdivided into perioperative and postoperative complications [5]. The most common perioperative complications are bad splits, bleeding, soft-tissue damage, dental damage, and temporomandibular-joint disturbances. Infections, bleeding, and neurosensory loss are among the common post-operative complications [3]. The frequencies of the above-mentioned complications are low, varying between 1.2 and 32% depending on study criteria [3, 4, 6]. Likewise, fatal outcomes are rare, and only a few case reports exist [7].

The aim of this paper is to report our experience with multiple complications following orthognathic surgery and the subsequent treatment to restore function and aesthetics in a female patient.

## Case presentation

**Fig. 1:**
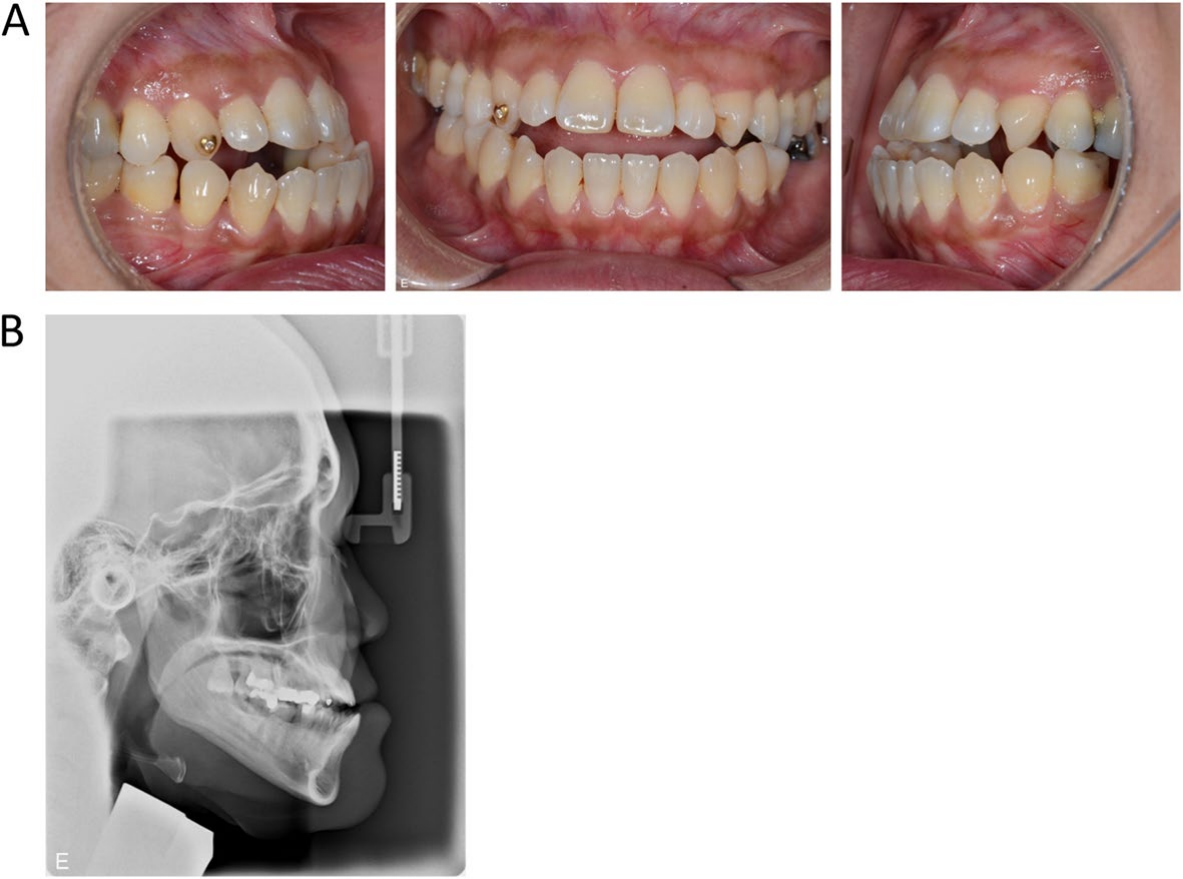
Intra-oral clinical photos showing a pre-operative frontal and sagittal view (**A**). Pre-operative left profile (**B**)

A female patient, born in 1975, was referred to the department of Oral and Maxillofacial Surgery, Norrlands University Hospital, Umeå, Sweden in October 2014. Her facial growth had resulted in sagittal and vertical discrepancy, resulting in a negative overjet and overbite of 3 mm (Fig. 1). Due to multiple molar losses, occlusal deterioration had caused masticatory problems. A combination therapy with orthodontics and orthognathic surgery was chosen. The aim was to normalize the patient’s basal and dental relationships between the jaws, resulting in improved occlusion and masticatory function. The surgical plan included a segmented LF I and BSSO.

During spring 2015 the pre-surgical orthodontic therapy started. A space distal to the maxillary canines was created that made it possible to perform a segmented LF I for correction of the vertical discrepancy. During the pre-surgical orthodontic treatment, the patient was diagnosed with bilateral maxillary sinusitis and hence treated saline rinsing and steroid nasal spray prescribed by ear-nose and throat (ENT) specialists. Due to the sinusitis, changes in responsible orthodontist and the fact that the patient travelled abroad for a longer period, the orthodontic treatment was prolonged.

In the end of February 2018, a physical medical examination was performed, and the patient was perceived as healthy. During the pre-surgical examination there were no indications of impaired healing capacity. The patient had no allergies or regular medications. She had undergone several tooth extractions without any post-operative complications. Virtual planning with IPS CaseDesigner Version 2.0 – KLS Martin resulted in a final plan with a bimaxillary surgical procedure with maxilla-first surgery followed by a BSSO with mandibular setback.

**Fig. 2:**
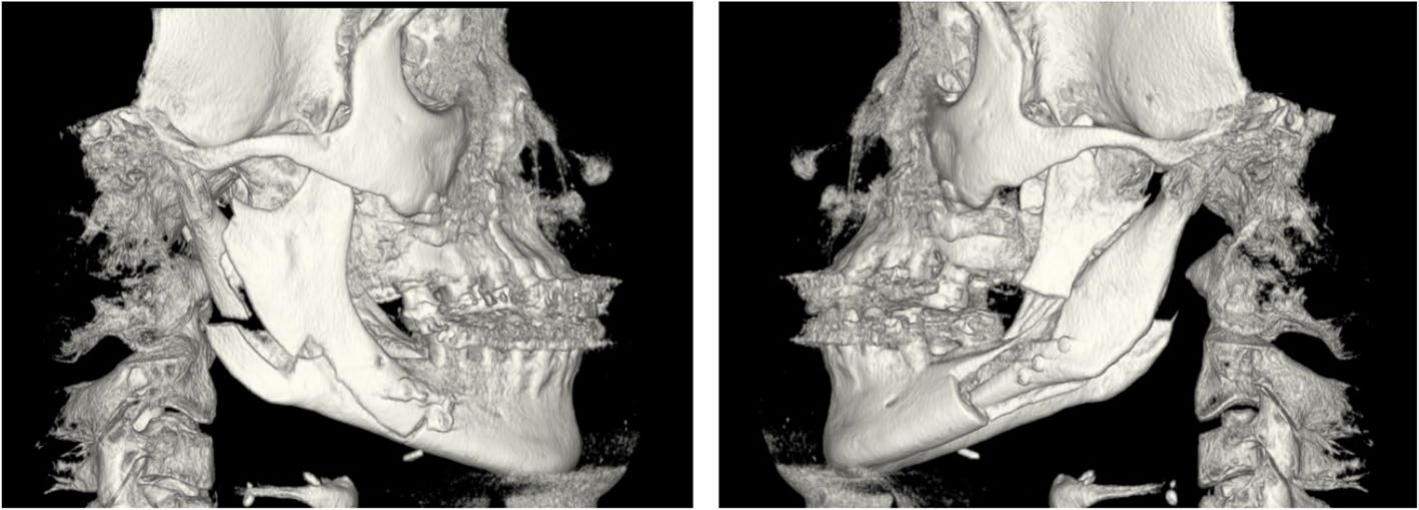
3D reconstructions made from the post-operative CBCT scan, showing the bilateral bad splits

**Fig. 3:**
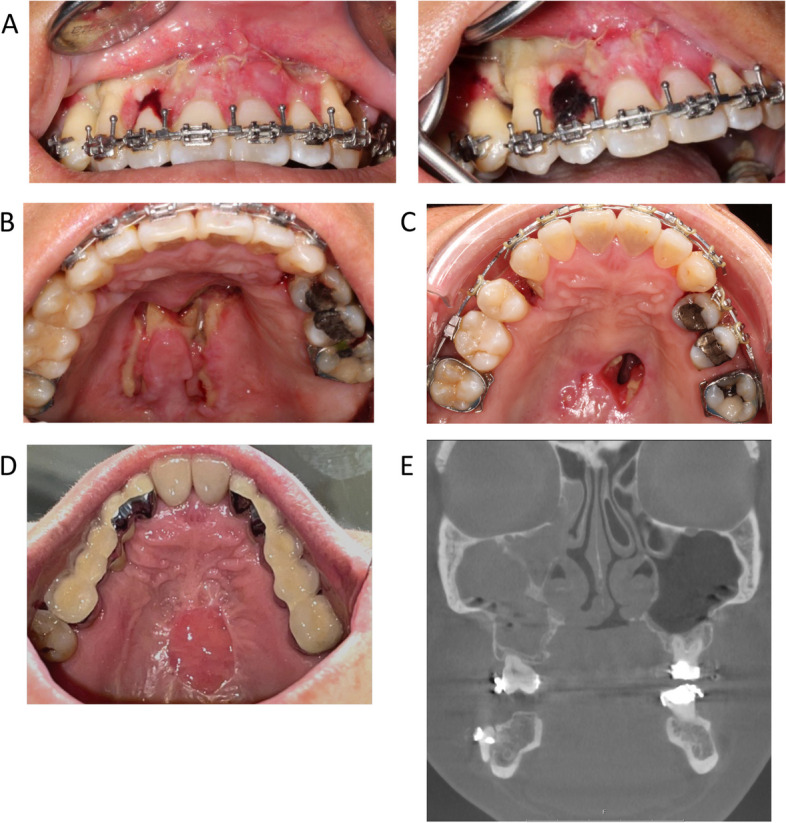
Intra-oral clinical photos showing (**A**) the soft tissue ischaemia in the vestibular gingiva, extending from tooth 13 to tooth 23. (**B**) The oro-nasal communications 7 days post operation, (**C**) 2 months post operation, and (**D**) restored soft tissue of the palate 36 months post operation. (**E**) A representative radiographic coronal view of the patient’s sinuses exhibiting clear signs of sinusitis on the right side

In the beginning of May 2018, the surgical procedure was performed by two oral and maxillofacial surgeons. The patient was under general anaesthesia with induced hypotension, intravenous peri-operative antibiotics (3 g benzylpenicillin), and haemostatic treatment (1 g tranexamic acid). Soft tissue incisions were performed with electrocuting, and all osteotomies were performed with a piezo saw and completed with osteotomes and spreaders. During mobilization of the down-fractured maxilla, the descending palatine arteries bilaterally exhibited severe tension and were ligated. A small soft-tissue lesion in the hard palate tissue was identified and sutured after fixation of the segmented maxilla in the new position. During BSSO, bilateral bad splits occurred (Fig. 2), but the situation could be remedied with additional osteotomies and fixation screws. The total peri-operative blood loss was 350 ml. The patient was discharged the first day after surgery with analgesics (paracetamol and NSAIDs) and antibiotics (fenoximethylpenicillin, 1.6 g × 3) for 10 days. At the 10-day post-operative follow-up appointment, an intense odour was detected, and signs of soft tissue ischaemia/necrosis were noticed in the vestibular gingival regions 13–23 (Fig. 3). Hence, gentle cleaning and prolonged antibiotics were recommended. Seven days later, an oro-nasal communication in the palate had appeared (Fig. 3). A splint covering the hard palate reduced the fluid leakage to the nasal cavity. During the first 6 postoperative weeks, necrosis of the vestibular marginal gingiva and alveolar bone supporting the first tooth on each side of the osteotomy occurred. The bone necrosis led to pulp necrosis in teeth 13/23 and resulted in endodontic treatment. The fixed appliance in the maxilla was kept for stabilization of the premaxilla 18 months postoperatively. The patient suffered from repeated bilateral maxillary sinusitis demanding antibiotics until teeth 13, 15, 23, and 24 were removed together with necrotic alveolar bone in October 2020. The extracted teeth were replaced with temporary bridges (regions 16–12, 22–26). The closure of the fistula in the palate required three surgical interventions between December 2019 and June 2021 (Fig. 3). The first and second attempt to close the fistula was performed by a plastic surgeon. During the first surgery a local vascularized buccal flap was used, rendering in a decreased fistula area. The following surgery was a local flap from a single side of the palatal mucosa. However, the fistula was not fully closed. The third attempt was a co-operation between a plastic surgeon specialized in cleft surgery together with the responsible oral and maxillofacial surgeon. Local flaps from both sides of the palate were used.

**Fig. 4:**
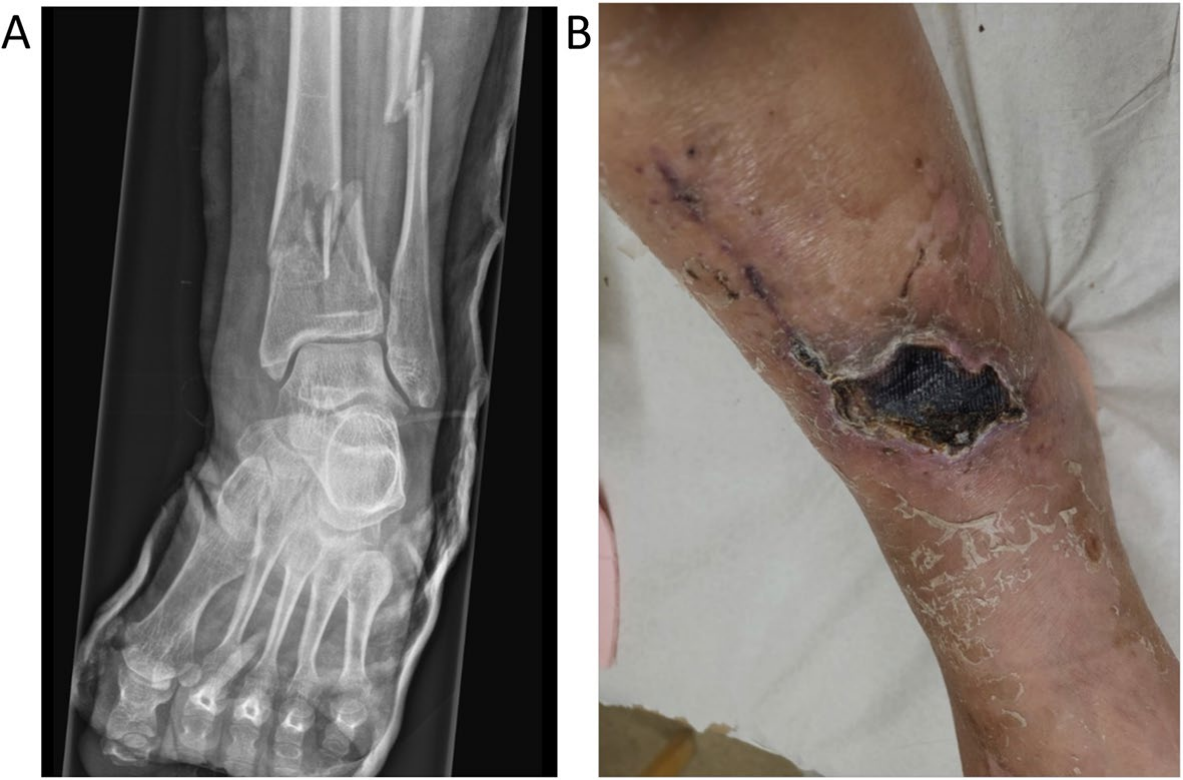
Radiograph showing the patient’s fractures of the left distal fibula and tibia (**A**). Unhealed soft tissue of the left leg 10 months after surgery (**B**)

In January 2022 the patient slipped in the snow and suffered of fractures of her left distal fibula and tibia. She was treated at the Orthopaedic Department at Norrlands University Hospital.. Open orthopaedic fixation was performed. The healing process of the soft tissue around the incisions did not progress as expected. After 10 months, the soft tissue of the patients’ left leg had still not fully recovered (Fig. 4).

**Fig. 5:**
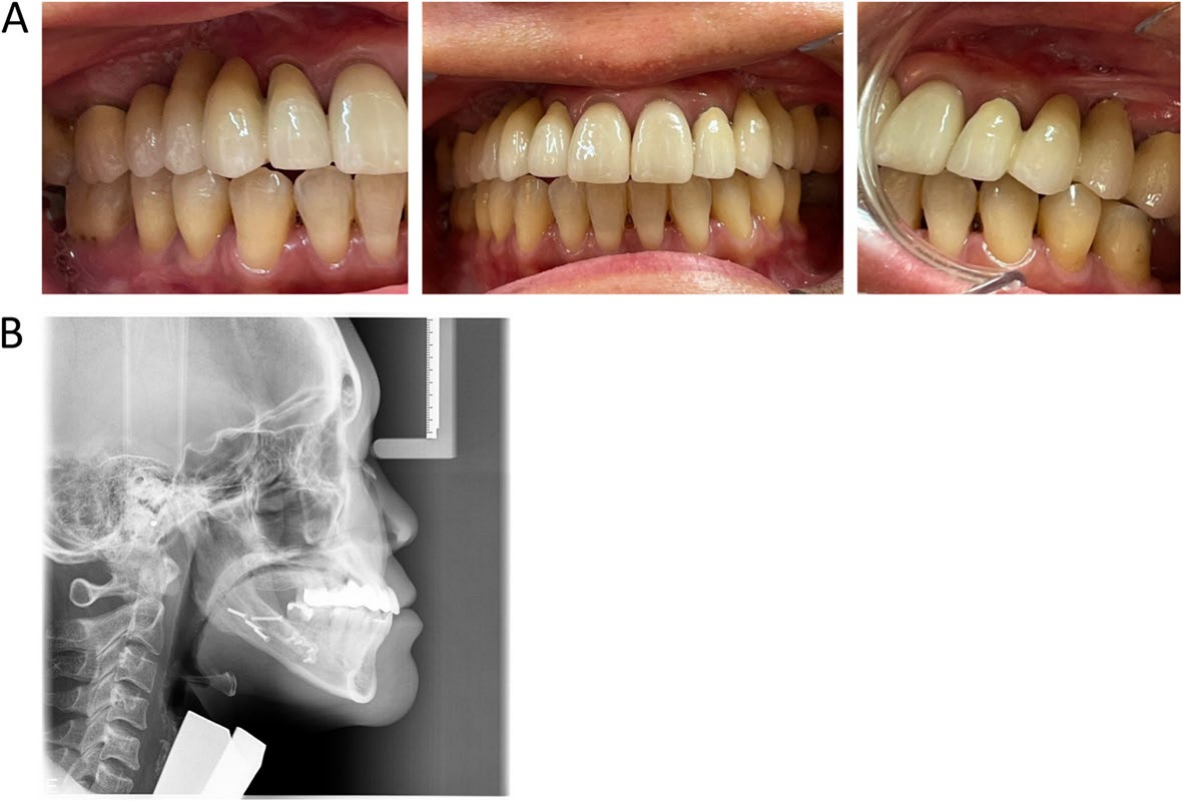
Intra-oral clinical photos showing a frontal and sagittal view 4 years post operation (**A**). Post-operative left profile (**B**)

In June 2022, 4 years after the orthognathic surgery, the patient was finally restored with fixed dental bridges in the maxilla (Fig. 5). The bone healing in the mandible was normal, despite the bad splits, but sensory function was still impaired in the left mandible.

## Discussion and conclusions

This case illustrates a unique, and to one person concentrated, variety of complications that may occur as a result of orthognathic surgery. Although the reasons for complications generally cannot be deduced from the literature, some data suggest that the risk of complications could be higher in individuals such as our patient who undergo bimaxillary surgery [8]. One can also speculate about the age of the patients for some of the complications that are described in literature. Here we discuss each complication for this unique orthognathic case..

### Ischaemia and necrosis

In Le Fort I osteotomies there is a small risk of avascular necrosis of the maxilla. The reported operative risk factors are, foremost, segmental osteotomies, large transverse expansions, anterior advancements of 9–10 mm, inadequate surgical techniques, intraoperative haemorrhage, and perforation/laceration of the palatal soft tissue [9]. In a prospective study regarding intra- and peri-operative complications of Le Fort I surgery, performed by Kramer et al., ischaemic complications affected 1.0% of the patients, including 0.2% who experienced an aseptic necrosis of the alveolar process [10, 11]. In our case the maxilla was segmented, and a perforation of the soft palate occurred. It has been suggested that segmentation of the maxilla results in greater risk of necrosis compared to non-segmented. However, new evidence show that there are no significant differences in complication rate between segmented or non-segmented Le Fort I [12], Furthermore, during surgery the descending palatine artery was ligated bilaterally, an intervention that may have reduced the blood supply to the maxilla. On the other hand, Dodson et al. performed a study on 34 patients with ligation of the descending palatine artery during Le Fort I osteotomy in 16 patients and compared them with 18 patients without ligation. The authors found no difference in maxillary gingival blood flow [13]. Taken together, the maxillary segmentation, perforation of the soft palate, and ligation of the palatine arteries in our patient could have contributed to the impaired healing of the maxilla, resulting in post-operative necrosis.

### Non-union of the osteotomy segments

The incidence of non-union of the segments is rare and has been reported to be 2.6% in a retrospective study containing 150 individuals [14]. It is more common in women than in men. The reported non-unions all appeared in individuals undergoing surgery to correct a class III bite [14]. In the systematic review by Jedrzejewski et al., the incidence is a bit higher, 4.55%, but not discussed in greater detail [5]. All the above-mentioned factors match our patient, and in combination with the severe necrosis constitute a potential explanation for the non-union of the osteotomy segments.

### Palatal fistula

As mentioned earlier, there was a rift in the soft tissue of the palate, which did not heal properly and gave rise to palatal fistula/oro-nasal communication. A retrospective study of segmented Le Fort I osteotomies reported that 2 individuals out of 262 developed a persistent oronasal fistula [15], indicating that it is a rare complication. The fistula was first covered by a splint covering the hard palate. The splint prevented fluid from the oral cavity to the nasal cavity, but the fistula did not heal. The reason was probably because necrotic bone in the hard palate was exfoliated. As mentioned in the case presentation three surgical attempts were made prior to complete closure and it might be hypothesized that local flaps from both sides from the palate is the most successful intervention to close oroantral fistulae.

### Maxillary sinusitis

The incidence of maxillary sinusitis after Le Fort I surgery is reported to be 4.76% [16]. There is also some evidence that Le Fort I osteotomies result in worsening of pre-operative maxillary sinusitis symptoms both radiologically and subjectively [17]. Our patient was treated pre-operatively for her sinusitis. During the surgery the maxilla was down fractured exposing a healthy sinus membrane without purulent fluid. The necrotic alveolar process in the osteotomy lines was probably one reason for the postoperative sinusitis. After removal of the necrotic alveolar bone the sinusitis subsided.

The healing of the fistula and the subsided sinusitis is probably the result of removal of necrotic bone.

### Bad split in the bilateral sagittal split osteotomy (BSSO)

‘Bad split’ refers to the unwanted or unfavourable pattern of the split osteotomy. The incidence is reported to vary between 0.21 and 22.7% [18]. Steens et al. demonstrated a significant but weak correlation between increasing age and bad split [19]. The patient presented in this case report can be considered older. However, age alone is not a robust explanation for the bilateral bad splits.

### Neurosensory loss

Neurosensory loss is a well-known adverse effect, and the incidence of permanent damage has been reported to be as high 21.7% per SSO and 33.9% per patient [20]. It has also been reported that age is probably the most important patient-related risk factor for having a persistent nerve damage [21]. One could argue that the patient experienced a permanent neurosensory loss on the left side of her mandible due to the bad split; however, there is no evidence that bad splits enhance the risk of neurosensory loss [20].

## Conclusion

This case clearly shows the importance of frequent clinical follow-ups with a multidisciplinary approach and openness to therapy changes during the post-operative period in situations with post-operative complications after orthognathic surgery. Moreover, one interesting fact is the soft-tissue dehiscence on her left lower leg that followed the orthopaedic surgery. One can speculate about an undiagnosed soft-tissue defect necessitating further analyses. The patient had no signs of impaired general health and no regular medication. Earlier surgical interventions in the oral cavity had a normal healing pattern. Due to the latest years clinical findings of impaired healing of foremost soft tissue we referred the patient to her general practitioner for further medical investigation.

## Data Availability

The datasets generated and analysed during the current study are not publicly available due to Swedish journal act but are available from the corresponding author on reasonable request.

## References

[1] Andrup ME (2015). Jesper; Ramirez, Eusebio; Sjöström, Mats, indications and frequency of Orthognathic surgery in Sweden – a questionnaire survey. Int J Oral Dental Health..

[2] Venugoplan SR (2012). Discharge patterns of orthognathic surgeries in the United States. J Oral Maxillofac Surg..

[3] Ferri J (2019). Complications in orthognathic surgery: a retrospective study of 5025 cases. Int Orthod..

[4] Panula K, Finne K, Oikarinen K (2001). Incidence of complications and problems related to orthognathic surgery: a review of 655 patients. J Oral Maxillofac Surg..

[5] Jedrzejewski M (2015). Preoperative, intraoperative, and postoperative complications in orthognathic surgery: a systematic review. Clin Oral Investig..

[6] Steel BJ, Cope MR (2012). Unusual and rare complications of orthognathic surgery: a literature review. J Oral Maxillofac Surg..

[7] Hwang K, Kim HJ, Lee HS (2013). Airway obstruction after orthognathic surgery. J Craniofac Surg..

[8] Kantar RS (2019). Bimaxillary Orthognathic surgery is associated with an increased risk of early complications. J Craniofac Surg..

[9] Ettinger KS (2020). Microvascular reconstruction of Total maxillary avascular necrosis as a complication of routine Orthognathic surgery. J Oral Maxillofac Surg..

[10] Kramer FJ (2004). Intra- and perioperative complications of the LeFort I osteotomy: a prospective evaluation of 1000 patients. J Craniofac Surg..

[11] Robl MT, Farrell BB, Tucker MR (2014). Complications in orthognathic surgery: a report of 1,000 cases. Oral Maxillofac Surg Clin North Am..

[12] Joseph MM (2023). Association between maxillary segmentation and perioperative complications in Le fort I osteotomy. J Craniofac Surg..

[13] Dodson TB, Bays RA, Neuenschwander MC (1997). Maxillary perfusion during Le fort I osteotomy after ligation of the descending palatine artery. J Oral Maxillofac Surg..

[14] Imholz B (2010). Non-union of the maxilla: a rare complication after Le fort I osteotomy. Rev Stomatol Chir Maxillofac..

[15] Posnick JC, Adachie A, Choi E (2016). Segmental maxillary osteotomies in conjunction with Bimaxillary Orthognathic surgery: indications - safety - outcome. J Oral Maxillofac Surg..

[16] Pereira-Filho VA (2011). Incidence of maxillary sinusitis following Le fort I osteotomy: clinical, radiographic, and endoscopic study. J Oral Maxillofac Surg..

[17] Nocini PF (2016). Is Le fort I osteotomy associated with maxillary sinusitis?. J Oral Maxillofac Surg..

[18] Chrcanovic BR, Freire-Maia B (2012). Risk factors and prevention of bad splits during sagittal split osteotomy. Oral Maxillofac Surg..

[19] Steenen SA, van Wijk AJ, Becking AG (2016). Bad splits in bilateral sagittal split osteotomy: systematic review and meta-analysis of reported risk factors. Int J Oral Maxillofac Surg..

[20] Verweij JP (2016). Risk factors for common complications associated with bilateral sagittal split osteotomy: a literature review and meta-analysis. J Craniomaxillofac Surg..

[21] Nesari S, Kahnberg KE, Rasmusson L (2005). Neurosensory function of the inferior alveolar nerve after bilateral sagittal ramus osteotomy: a retrospective study of 68 patients. Int J Oral Maxillofac Surg..

